# Detecting and Diagnosing Atrial Fibrillation (D_2_AF): study protocol for a cluster randomised controlled trial

**DOI:** 10.1186/s13063-015-1006-5

**Published:** 2015-10-23

**Authors:** Steven B. Uittenbogaart, Nicole Verbiest-van Gurp, Petra M. G. Erkens, Wim A. M. Lucassen, J. André Knottnerus, Bjorn Winkens, Henk C. P. M. van Weert, Henri E. J. H. Stoffers

**Affiliations:** Department of General Practice, Amsterdam Medical Center, J2–118, PO Box 22660, 1100 DD Amsterdam, The Netherlands; Department of Family Medicine, School for Public Health and Primary Care (CAPHRI), Maastricht University, PO Box 616, 6200 MD Maastricht, The Netherlands; Department of Methodology and Statistics, School for Public Health and Primary Care (CAPHRI), Maastricht University, PO Box 616, 6200 MD Maastricht, The Netherlands

**Keywords:** Atrial fibrillation, Case-finding, ECG, Pulse palpation, Elderly population, Primary care setting, General practitioner, Stroke, Cardiovascular risk

## Abstract

**Background:**

Atrial fibrillation is a common cause of stroke and other morbidity. Adequate treatment with anticoagulants reduces the risk of stroke by 60 %. Early detection and treatment of atrial fibrillation could prevent strokes. Atrial fibrillation is often asymptomatic and/or paroxysmal.

Case-finding with pulse palpation is an effective screening method, but new methods for detecting atrial fibrillation have been developed. To detect paroxysmal atrial fibrillation ambulatory rhythm recording is needed.

This study aims to determine the yield of case-finding for atrial fibrillation in primary care patients. In addition, it will determine the diagnostic accuracy of three different case-finding methods.

**Methods/Design:**

In a multicenter cluster randomised controlled trial, we compare an enhanced protocol for case-finding of atrial fibrillation with usual care. We recruit 96 practices. We include primary care patients aged 65 years or older not diagnosed with atrial fibrillation. Within each practice, a cluster of 200 patients is randomly selected and marked. Practices are evenly randomised to intervention or control group. The allocation is not blinded.

When a marked patient visits an intervention practice, the case-finding protocol starts, consisting of: pulse palpation, sphygmomanometer with automated atrial fibrillation detection and handheld single-lead electrocardiogram (ECG). All patients with at least 1 positive test and a random sample of patients with negative tests receive a 12-lead ECG. Patients without atrial fibrillation on the 12-lead ECG, undergo additional continuous Holter and use the handheld single-lead ECG at home for 2 weeks.

Control practices provide care as usual.

The study runs for 1 year in each cluster. The primary outcomes are the difference in detection rate of new AF between intervention and control practices and the accuracy of three index tests to diagnose AF. We are currently recruiting practices.

The ‘Detecting and Diagnosing Atrial Fibrillation’ (D_2_AF) study will determine the yield of an intensive case-finding strategy and the diagnostic accuracy of three index tests to diagnose atrial fibrillation in a primary care setting.

**Trial registration:**

Netherlands Trial Register: NTR4914, registered on the 25 of November 2014.

## Background

Atrial fibrillation (AF) is a common cardiac arrhythmia, associated with substantial health risks. AF increases mortality, reduces the quality of life and increases the risk of heart failure. Moreover, AF is an important risk factor for stroke. Prevalence of AF increases with age, from 1 % in the general population up to 7–8 % in people over 65 years old [1, 2]. With increasing age, patients also have a higher risk of stroke [3]. Up to 30 % of ischemic strokes are AF-related [4–6]. Treatment with oral anticoagulants reduces the risk of stroke by 60 % [7, 8]. However, in nearly a quarter of patients with stroke AF is detected after this event [9]. If we can detect AF before a stroke occurs and start anticoagulant therapy when appropriate, we may be able to prevent more than half of AF-related strokes [4, 6, 9–11].

Although AF can be a symptomatic condition with patients experiencing palpitations, exercise intolerance or fatigue, AF often is asymptomatic [12] and remains undetected. Furthermore, patients can have recurrent intermittent episodes of AF, paroxysmal AF (pAF). As with persistent AF, the risk of stroke is substantially increased in patients with pAF [13, 14]. Therefore, detecting pAF is as important as detecting persistent AF, but more challenging.

To establish the diagnosis of AF, an electrocardiogram (ECG) recording – either on a regular 12-lead ECG or a 30-second rhythm strip – has to be made showing irregular RR intervals without distinct P waves. When used as screening tools, ECG and ambulatory rhythm recording are costly and time-consuming. Case-finding in general practice using pulse palpation, improved the detection of AF compared with care as usual (incidence of AF 1.63 % versus 1.04 %, difference 0.59 %, 95 % CI 0.20 % to 0.98 %) [15]. Opportunistic screening in patients aged 65 years and over was as effective as systematic screening but at lower costs [15].

Recently, new methods for detecting AF have been developed [16]. One development is the equipment of automatic sphygmomanometers with an algorithm for irregular beat detection (eBPM-AF). The National Institute for Health and Clinical Excellence (NICE) guideline advocates the use of one of these devices, i.e. the Microlife® WatchBP Home A device [17]. Other methods use a single-lead ECG. The MyDiagnostick (Applied Biomedical Systems® (ABS), Maastricht, The Netherlands) is an example of a handheld ECG device, which records a single-lead ECG (left-right arm) [18].

To detect cases of pAF, ambulatory rhythm recording is needed [19]. Traditionally, either 24-hour Holter or event recording is used. In post-stroke patients, a 30-day period of automatically triggered event recording increased the detection rate of pAF 5-fold compared with a 24-hour Holter (detection rate of 16.1 % versus 3.2 %) [20]. In primary care patients with palpitations, monitoring for 14 days diagnosed about 80 % of relevant arrhythmias [19].

New developments have enabled the use of continuous ECG registrations for longer periods (continuous Holter) and make loop recording unnecessary. In general, ambulatory rhythm recording is used in symptomatic patients or as part of follow-up after stroke. There is insufficient evidence about the effectiveness of ambulatory rhythm registration for the detection of AF in asymptomatic patients (in general practice). This is a major evidence gap and further research is recommended [21, 22].

The ‘Detecting and Diagnosing Atrial Fibrillation’ (D_2_AF) study investigates whether enhanced opportunistic case-finding – using pulse palpation, single-lead handheld ECG and eBPM-AF – increases detection of AF in general practice patients of 65 years and over. Additionally, the study determines the most effective method of case-finding by comparing the diagnostic yield of pulse palpitation, handheld ECG and eBPM-AF alone and in combination.

## Methods

### Design

**Fig. 1:**
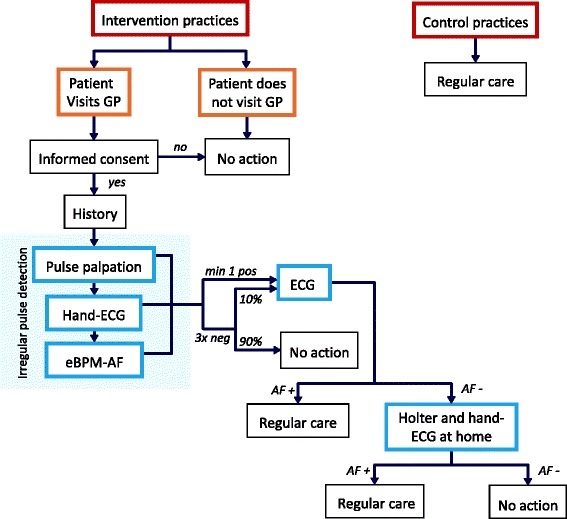
Study design of ‘Detecting and Diagnosing Atrial Fibrillation’ D_2_AF. GP, general practitioner; hand-ECG, handheld ECG device; eBPM-AF, automatic sphygmomanometer with an algorithm for irregular beat detection; ECG, electrocardiogram

We perform a multicenter cluster randomised controlled trial (RCT) comparing enhanced case-finding of AF with usual care. In each practice, the study runs for 1 year. In the intervention arm, we run a diagnostic study comparing 3 different methods for case-finding of AF with a composite reference standard consisting of a 12-lead ECG and a 2 week Holter. This way, we determine the yield of each method individually and combined to detect unknown AF. Figure 1 shows a flowchart of the study design.

### Ethics

The D_2_AF study proposal was approved by the medical ethical board of the Academic Medical Center in Amsterdam (dated 14 November 2014, number NL48215.018.14). The D_2_AF study is registered at The Netherlands Trial Register (NTR number NTR4914).

### Participants: practices and patients

General practices are recruited across The Netherlands. We regard two or more general practices sharing facilities as one practice. Within each practice, we form one cluster by selecting a random fixed sample of two hundred patients out of the eligible patients.

The inclusion criterion for selection of the cohort is an age of 65 years and over; the exclusion criterion is previously documented AF. Additional criteria are applied only to patients in the intervention group for whom case-finding would be inappropriate (see intervention practices). After selection of the study cohort, we extract the medical files to determine the baseline characteristics (e.g. age, sex and medical history including heart disease, hypertension, previous stroke/transient ischaemic attack (TIA), diabetes).

All data are entered into our secured trial management system. This system also gathers all other data during the study. Investigators enter data directly into the system using electronic case record forms (eCRF).

### Cluster randomisation

Clusters are evenly allocated to either the intervention or the control group using stratified randomisation. To stratify clusters, we determine the prevalence of AF in all patients aged 65 years and over in participating clusters using International Classification of Primary Care (ICPC)-codes in the medical files. Subsequently, we stratify clusters in high-prevalence and low-prevalence clusters. As a cut-off we use the average prevalence of AF in the associated general practices of the 2 universities involved in this study: 8.05 %. We use computerised randomisation in permuted blocks of random sizes. After randomisation, the investigator informs the participating practices of the allocation.

### Intervention practices

In each practice, the selected 200 patients are marked in the medical records of the practice before the start of the study. When a marked patient makes an appointment for a consultation at the practice we ask him or her to participate. The sub-investigator (practice nurse, practice assistant or general practitioner) does not carry out the intervention in patients not suited for case-finding based on the following criteria:

- having a pacemaker or implantable cardioverter-defibrillator (ICD)
- legal incompetence or inability to give informed consent
- suffering from a terminal illness (as defined by the general practitioner)
- inability to come to the practice to participate in the diagnostic process; for instance, a patient who is chronically bedridden. Patients who cannot visit the practice due to a temporary situation (such as the flu) are not excluded

The reason is noted in the trial management system. All patients who undergo the case-finding protocol in the intervention group will be asked written informed consent. We do not ask informed consent in the control group since we merely analyse these patients on a group level. They receive care as usual and we do not want to trigger additional awareness of AF.

#### Diagnostic tests

The sub-investigator collects the baseline information on the patient history, weight, length and current symptoms indicative of AF. Subsequently, the sub-investigator performs the following three index tests in all study patients:

*Pulse palpation*: manual palpation of the radial artery in the wrist with the fingertips for a minimum of 15 seconds. The frequency is registered. The heart rhythm is classified as ‘regular’, ‘one to three skipped or extra beats’ or ‘irregular’. Both ‘one to three skipped or extra beats’ and ‘irregular’ are regarded as a positive result. The filling pressure is classified as ‘equal’ or ‘unequal’.

*eBPM-AF*: the WatchBP Home A (Microlife®, Widnau, Switzerland) is an electronic sphygmomanometer with an algorithm for irregular beat detection. The algorithm calculates the irregularity index based on the interval time between heartbeats and indicates an irregular pulse if the threshold is exceeded. A cuff is applied around the patient’s left or right upper arm. The cuff inflates and deflates automatically after pressing the ‘ON’ button. The display shows the average of three blood pressure measurements. If an irregular pulse is detected in at least two out of three measurements, an icon on the display saying ‘Afib’ starts blinking [17]. If the device is not able to correctly analyse the rhythm this is noted in the eCRF.

*Handheld ECG at the practice*: the MyDiagnostick (ABS, Maastricht, The Netherlands) is a 24-cm long bar with metallic electrodes at both ends. It records a single-lead ECG (left-right arm). The device switches on automatically when holding it with both hands. An automatic algorithm calculates a rhythm-score, periodicity-score and variability-score based on computed intervals between two R waves. If the threshold is exceeded, a red light indicates possible AF. The outcome of the handheld ECG is noted in the eCRF. If the device cannot make a recording this is registered. Recordings are stored locally and are uploaded as a PDF in the local PC application [23].

The order in which the index tests are performed is set. Pulse palpation is performed and recorded first. Then, by an alternating pre-set order in the eCRF, both eBPM-AF and handheld ECG are performed and recorded.

All patients scoring positive on at least 1 of the 3 index tests will receive a 12-lead ECG in the same session as the index tests. To calculate sensitivity and specificity of the screening procedure, a random sample of 10 % of patients who score negative on all 3 index tests also receive a 12-lead ECG. An overview of these procedures can also be seen in Fig. 1. The sub-investigator is blinded to the ECG results while performing the index tests.

12-lead ECG: the 12-lead ECG device (Fysiologic®, Amsterdam, The Netherlands) is paperless and does not display the ECG. The ECG is transferred digitally and assessed by an experienced assessor, supervised by a cardiologist. In case the ECG shows any serious clinically relevant abnormality, the general practitioner (GP) is notified immediately. A second cardiologist re-assesses all AF-diagnosed ECGs and a random sample of negative ECGs. Both cardiologists are provided with basic data such as age and gender but are blinded to all previous measurements. Any disagreement on diagnosis is solved by a third cardiologist. AF is defined as the absence of distinct P waves and a completely irregular RR interval [24].

If a patient is diagnosed with AF, the study protocol is finished. Results of the ECG are reported back to the GP, who provides care at his discretion.

In patients in whom no AF is diagnosed on a 12-lead ECG, 2 additional tests are performed to detect pAF:

- Handheld ECG at home: the same device (MyDiagnostick, ABS, Maastricht, The Netherlands) as mentioned above is used, but the indication light is set off. Thus, the patient is blinded for the results of the test (light will not blink). The patient is instructed to use the handheld ECG three times a day at set times. After 2 weeks of recording, data are uploaded as PDF in the local PC-application. The MyDiagnostick can store up to 140 ECG strips.
- Two-week Holter: the same device (Fysiologic®, Amsterdam, The Netherlands) as for the 12-lead ECG is used. The wiring has four different patches instead of ten. With these 4 patches, leads V1 and V5 are obtained. The patient receives a small disposable shoulder bag to wear the device and several sets of patches with instructions so they can change them. After 2 weeks of continuous recording, data are transferred and assessed by an experienced assessor, supervised by a cardiologist. The number and duration of AF episodes are registered. In case the ECG shows any serious clinically relevant abnormalities, the GP is notified immediately. A second cardiologist, blinded to previous assessments re-assesses all AF-diagnosed Holters. Furthermore, a random sample of negative Holters is re-assessed. We define AF as any arrhythmia that has the characteristics of AF (see above) and lasts at least 30 seconds [24].

#### Diagnoses ‘atrial fibrillation’

To diagnose AF, we use a composite reference standard consisting of the results of the 12-lead ECG and the 2-week Holter as defined previously.

Besides cases found with our case-finding protocol, we will also perform computer searches of the medical records to identify all new cases of AF. We ask the sub-investigator to report how the diagnosis was established in new cases of AF not detected with the intervention.

#### Quality of life

Little is known of the quality of life of asymptomatic AF patients. To investigate this, we use the EuroQol 5D (EQ-5D) questionnaire to measure quality of life. EQ-5D is a generic tool that assesses five dimensions of health-related quality of life by use of a questionnaire and a visual analogue scale [25]. We opt for 1000 patients in the intervention practices to participate: all patients with newly diagnosed AF saturated with a random sample of patients not diagnosed with AF. The reason for the visit may affect quality of life at the time of the visit. Therefore, we delay the timing of the questionnaire until the end of the study year.

### Control practices

In each practice, the selected 200 patients are marked in the medical records of the practice before the start of the study, but the marking is blinded to the sub-investigators. Neither the sub-investigators nor the patients are aware of participation in the study. The control practices perform ‘usual care’ at the discretion of the GP. Currently, the guideline on AF of the Dutch College of General Practitioners does not recommend screening for AF. Pulse palpation is not carried out systematically.

At the end of the study year, we perform computer searches of the medical records to identify all new cases of AF in the control practices. We evaluate the route that led to the diagnosis of AF. To that end, we will study the patients’ files to determine the path by which the diagnosis AF is made.

### End of the study

The study ends when all clusters have finished their 1-year study period.

### Outcome measures

#### Primary outcome measures

1. The yield of a case-finding strategy for AF (including paroxysmal and asymptomatic AF) compared with care as usual.
2. The diagnostic accuracy (sensitivity and specificity) of each of the three index tests (pulse palpation, eBPM-AF and handheld ECG) to diagnose AF and the accuracy of various combinations of case-finding strategies with the results of the 12-lead ECG and 2-week Holter as reference standard.

### Secondary outcome measures

1. The incidence and prevalence of AF (including paroxysmal and asymptomatic) in patients aged 65 years and over in Dutch general practice.
2. The diagnostic accuracy (sensitivity and specificity) of the handheld ECG at home to detect pAF with the 2-weeks Holter as the reference standard.
3. The quality of life of patients with newly detected asymptomatic AF compared with healthy controls as measured with the EQ-5D.

### Sample size calculation

The primary outcome is the difference in the detection rate of new AF in 1 year between intervention and control practices. We estimate that the yearly incidence of new AF in the Netherlands in patients aged 65 and over is 1.3 % and assume that this is the detection rate in control practices [26]. In previous research among a similar group of patients the odds ratio (OR) of newly identified AF by opportunistic screening compared with no screening in the control group was 1.61 (95 % CI 1.14–2.29) [15]. In that study, ECG was offered if the pulse of the patient was irregular. Since we will provide a more extensive diagnostic programme of various diagnostic methods, it is likely that we will find a higher detection rate of (paroxysmal and asymptomatic) AF. We assume that an OR of 1.8 is reasonable. The detection rate in the intervention group is estimated at 2.32 % [27]. To detect an absolute difference of 1.02 % in the detection rate of new cases of AF between the intervention and control arm of the study with 80 % power at a significance level of 5 %, we will need 2701 patients in each group [28]. Outcomes for individuals within clusters may be correlated as we will randomise practices (i.e. clusters). To take into account the effect of cluster randomisation we calculate a design effect of 2.99 based on fixed cluster sizes of 200 patients and an intracluster correlation coefficient (ICC) of 0.01 [29, 30]. In a study comparable to ours, an ICC of 0.0027 between practices was found at the end of the study [15]. Increasing the sample size by a design effect of 2.99 the number of required inclusions becomes 8076 patients per group. Additionally, we take into account a loss to follow-up of 15 %. After correcting for loss to follow-up we get a total of 9501 patients. We decided on 48 even cluster sizes of 200 patients which makes a total of 9600 patients. Our sample size and study design are comparable to the study by Hobbs et al. [22].

### Statistical analysis

We analyse data on an intention-to-screen basis. We evaluate the outcomes of the intervention on a patient level.

### Primary study outcomes

1. The difference in yield of new AF in 1 year between intervention and control practices is estimated by calculating the difference in detection rate of new AF (new cases of AF after 1 year within the clusters/all patients in the clusters) between intervention and control practices. We use logistic mixed-effects models with practice as random factor for statistical analysis, since this accounts for the correlation between patients within the same practice. In case the outcome (AF yes/no) is missing for a patient, the patient is excluded from the analysis (list-wise deletion). We use multiple imputation to obtain complete datasets and to see whether the original analysis is influenced by these missing data: i.e. to see whether the difference in detection rates between intervention and control groups is similar for both the original analysis (list-wise deletion) and the multiple imputation analysis.
2. Diagnostic test characteristics of the different index tests to diagnose AF are compared with the results of the 12-lead ECG and 2-weeks Holter (composite reference standard). Sensitivity and specificity of each technique are calculated (including the corresponding 95 % confidence intervals). Because not all patients receive the reference standard, diagnostic parameters are estimated by converting studied numbers (i.e. results of test and reference standard) to the source population by multiplying them with the sample factor. For this, we use inversed probability weighting [31]. In the intervention group, we expect an incidence of AF of 2–3 %. This would result in sufficient numbers in all cells of the imaginary diagnostic 2x2 tables. We compare the diagnostic characteristics of the index tests to each other. We evaluate each method separately and in different combinations.

### Secondary study outcomes

1. We use descriptive statistics in both intervention and control practices to provide current AF prevalence and incidence figures in Dutch general practice.
2. The diagnostic test characteristics of the handheld ECG device for home monitoring using the 2-week Holter as reference standard are reported in a 2x2 table. Sensitivity and specificity (including the corresponding 95 % confidence intervals) are calculated.
3. We use descriptive statistics to describe the outcomes of the quality of life of patients with asymptomatic AF and healthy controls.

Categorical data are presented by number of patients (%) and numerical data by mean (SD) or median (interquartile range, IQR), where appropriate. IBM SPSS (SPSS Inc., Chicago, IL, USA) for Windows and SAS (SAS Institute, Cary, NC, USA) will be used to analyse the data.

## Discussion

The ‘Detecting and Diagnosing Atrial Fibrillation’ (D_2_AF) study is the first case-finding study to detect AF in general practice not only using pulse palpation but also new methods like a single-lead handheld ECG – to be used at the office and at the patient’s home – and an electronic sphygmomanometer with the ability to detect AF while measuring the blood pressure.

In a systematic review, Lowres et al. showed that screening for AF at one moment can identify previously undiagnosed AF [32]. The incidence of previously undiagnosed AF in patients of 65 years and over was 1.4 % (CI 1.2–1.6 %). However, when using pulse palpation or 12-lead ECG at only one moment, one risks missing pAF. A systematic screening programme in a 75-year-old Swedish population showed that 2 weeks of additional intermittent ECG recording using a single-lead ECG twice daily (after a negative 12-lead ECG) revealed newly found pAF in 7.4 % of screened patients [33]. The D_2_AF study uses new diagnostic methods and intermittent ECG recording to detect persistent and paroxysmal AF. By comparing the new methods (eBPM-AF and handheld ECG) and pulse palpation, we will be able to determine the diagnostic gain of each method separately and of combined strategies. Furthermore, we will determine the gain of using the handheld ECG at home for a prolonged period.

We use cluster randomisation to prevent control patients from being contaminated with awareness of AF detection or with the availability of additional equipment. We use a fixed sample size of 200 patients, as a compromise between statistical power and an acceptable workload for the general practices.

We focus on patients aged 65 years and over, because of the increasing prevalence of AF with age from about 1 % in the whole population to about 5 % in people aged over 65 [34]. Moreover, detecting AF in this age group has treatment consequences in most patients. According to the CHA_2_DS_2_VASc score all women and most men of 65 years and over with AF are eligible for anticoagulation. By recruiting in different regions, we include practices with diversity in organisation and patients characteristics, such as ethnicity.

Besides the actual prevalence in the patient group, the efforts the doctors made to detect AF in the past determines the registered prevalence of AF in each cluster. Therefore, we expect the registered prevalence of AF in each practice to correlate negatively with the chance of detecting new AF. We will use prevalence of AF in patients aged 65 years and older as our stratification variable to equalise the chance of finding new AF in the intervention and control practices.

We use a case-finding protocol, which means that only patients making an appointment for a consultation are asked to participate. We preferred this method to systematic screening, where all patients are asked to participate. One of the reasons is that Hobbs et al. showed case-finding to be as effective as systematic screening [15].

In the intervention arm, we run a diagnostic study to determine the test characteristics of different case-finding methods for AF. Ideally, one should perform the reference standard in all patients. We considered this as too costly, time-consuming and interruptive for both patients and practices. Therefore, we decided to perform the composite reference test only in patients with a positive case-finding test and a small random group of patients with negative tests.

AF is an important risk factor for stroke, and early detection is desirable to enable prevention of serious complications. The D_2_AF study will provide valuable evidence on the efficacy of case-finding and on the best methods for detecting AF.

## Trial status

The trial has started September 2015 in the first practices. We are currently completing the recruitment of practices.

## Abbreviations

AF: Atrial fibrillation
D_2_AF: ‘Detecting and Diagnosing Atrial Fibrillation’
eBPM-AF: Automatic sphygmomanometer with an algorithm for irregular beat detection
ECG: Electrocardiogram
eCRF: Electronic case report form
EQ-5D: EuroQol-5D
GP: General practitioner
ICC: Intracluster correlation coefficient
ICD: Implantable cardioverter-defibrillator
ICPC: International Classification of Primary Care
NICE: National Institute for Health and Clinical Excellence
OR: Odds ratio
pAF: Paroxysmal AF
RCT: Randomised controlled trial
TIA: Transient ischaemic attack
